# High Concentrations of Measles Neutralizing Antibodies and High-Avidity Measles IgG Accurately Identify Measles Reinfection Cases

**DOI:** 10.1128/CVI.00268-16

**Published:** 2016-08-05

**Authors:** Sun B. Sowers, Jennifer S. Rota, Carole J. Hickman, Sara Mercader, Susan Redd, Rebecca J. McNall, Nobia Williams, Marcia McGrew, M. Laura Walls, Paul A. Rota, William J. Bellini

**Affiliations:** National Center for Immunization and Respiratory Diseases, Division of Viral Diseases, Centers for Disease Control and Prevention (CDC), Atlanta, Georgia, USA; Vanderbilt University Medical Center

## Abstract

In the United States, approximately 9% of the measles cases reported from 2012 to 2014 occurred in vaccinated individuals. Laboratory confirmation of measles in vaccinated individuals is challenging since IgM assays can give inconclusive results. Although a positive reverse transcription (RT)-PCR assay result from an appropriately timed specimen can provide confirmation, negative results may not rule out a highly suspicious case. Detection of high-avidity measles IgG in serum samples provides laboratory evidence of a past immunologic response to measles from natural infection or immunization. High concentrations of measles neutralizing antibody have been observed by plaque reduction neutralization (PRN) assays among confirmed measles cases with high-avidity IgG, referred to here as reinfection cases (RICs). In this study, we evaluated the utility of measuring levels of measles neutralizing antibody to distinguish RICs from noncases by receiver operating characteristic curve analysis. Single and paired serum samples with high-avidity measles IgG from suspected measles cases submitted to the CDC for routine surveillance were used for the analysis. The RICs were confirmed by a 4-fold rise in PRN titer or by RT-quantitative PCR (RT-qPCR) assay, while the noncases were negative by both assays. Discrimination accuracy was high with serum samples collected ≥3 days after rash onset (area under the curve, 0.953; 95% confidence interval [CI], 0.854 to 0.993). Measles neutralizing antibody concentrations of ≥40,000 mIU/ml identified RICs with 90% sensitivity (95% CI, 74 to 98%) and 100% specificity (95% CI, 82 to 100%). Therefore, when serological or RT-qPCR results are unavailable or inconclusive, suspected measles cases with high-avidity measles IgG can be confirmed as RICs by measles neutralizing antibody concentrations of ≥40,000 mIU/ml.

## INTRODUCTION

Despite continued importations of measles virus into the United States, the elimination of indigenous measles has been maintained for over 15 years because of sustained high coverage with two doses of measles-mumps-rubella (MMR) vaccine (1–3). Many countries have eliminated measles or have made significant progress toward achieving goals for measles elimination (4). However, measles remains endemic in many parts of the world and both sporadic cases and large outbreaks have occurred in the United States following importations of the virus (5, 6). Although most measles cases in the United States have occurred among unvaccinated individuals, some confirmed cases have occurred among vaccinated and presumptively immune individuals (7, 8). In populations with high vaccination coverage, the number of susceptible individuals who are vaccinated will increase with time and will make up a larger proportion of the measles cases (9).

Laboratory confirmation of measles virus infection is a critical component of the surveillance required to support measles control and elimination programs. Though detection of measles virus-specific IgM by enzyme immunoassay (EIA) is the most widely used method to confirm measles virus infection, suspected measles cases in highly vaccinated populations may require additional testing. Inconclusive results obtained by IgM testing can be confirmed by detection of measles virus RNA by reverse transcription (RT)-PCR.

A suspected measles case in a previously vaccinated individual can be classified as a primary vaccine failure (PVF) by measurement of low-avidity measles IgG antibody (10). Individuals with confirmed measles and a prior immunologic response to measles virus (reinfection) from either vaccination or natural disease that occurred at least 4 months before symptom onset can be identified by the presence of high-avidity measles IgG antibody (10–13). A measles virus reinfection that occurs in an individual who had measurable specific antibodies after documented vaccination constitutes a secondary vaccine failure (SVF) (14–16). However, the vaccination history of some persons with confirmed reinfections can be unknown, and among those with ≥1 documented doses of vaccine, evidence of a protective titer of antibody to measles following vaccination is rarely available. Therefore, the term reinfection case (RIC) can be universally applied to a confirmed measles case in a person with high-avidity measles IgG antibody.

Serum samples collected at or near the onset of rash from RICs often have undetectable measles-specific IgM while high levels of measles-specific IgG are present (16–18). Therefore, the best method for case confirmation of a RIC is RT-PCR testing. However, reliable and dependable RT-PCR results depend on high-quality RNA extracted from specimens that have been adequately collected and transported to the laboratory in a timely manner. Because a good-quality specimen cannot be ensured, a negative RT-PCR result does not rule out a suspicious case. This may be especially problematic for RICs since the duration of viral shedding may be diminished and measles may not be initially suspected among those RICs with mild symptoms or unusual rash presentation and progression (18–21). However, measurement of high concentrations of measles neutralizing antibodies by the plaque reduction neutralization (PRN) assay, previously observed among confirmed measles cases with high-avidity measles IgG, may provide an alternative method to confirm RICs when standard laboratory tests are inconclusive (11, 22).

In this study, persons suspected of having measles with high-avidity measles IgG antibody in serum were tested by standard laboratory methods and were classified as having either a RIC or a rash illness not attributable to measles (noncase). Receiver operating characteristic (ROC) curve analysis was performed by using measles neutralizing antibody concentrations from these two groups as the discriminating variable to evaluate the use of neutralizing antibody concentrations to distinguish between RICs and noncases.

## MATERIALS AND METHODS

### Study samples and routine diagnostic methods.

Clinical samples from persons with suspected cases of measles included in this study were submitted to the Centers for Disease Control and Prevention (CDC) for routine confirmatory testing during 2006 to 2014. As part of routine surveillance, serum samples were tested for measles-specific IgM with the CDC measles IgM capture EIA and measles-specific IgG was detected with either the CDC measles IgG indirect EIA (23) or a commercially available measles IgG indirect EIA (Wampole Laboratories, Inc. Princeton, NJ, USA). Specimens for virus detection (throat swab, nasopharyngeal swab, and urine) were tested with a measles real-time RT-quantitative PCR (RT-qPCR) assay as previously described (24). In this group, there were 149 persons with suspected cases and positive results for measles-specific IgG in serum that were evaluated according to the criteria provided for inclusion in the ROC analysis (see below). Most of these samples were submitted from the United States; however, samples were also referred from the Federated States of Micronesia and the national laboratory in Colombia. Any case patients known to have received immunoglobulin as postexposure prophylaxis or noted to have a medical condition that could potentially diminish their immune response were excluded. In addition, patients who had received a postexposure dose of MMR vaccine were excluded unless infection with wild-type measles virus was confirmed by sequencing. Epidemiologic data associated with the suspected cases were compiled from the CDC specimen submission form or the National Notifiable Disease Surveillance System. Additional sources of information included telephone or email correspondence with epidemiologists or other public health staff involved in the investigation of the cases. The data collected included the setting in which the suspected case was identified (outbreak-associated or sporadic case), any known exposure or risk factors, known link to a secondary case, and vaccination (MMR vaccine) status.

### Terminology and criteria for inclusion in the ROC analysis.

**FIG 1:**
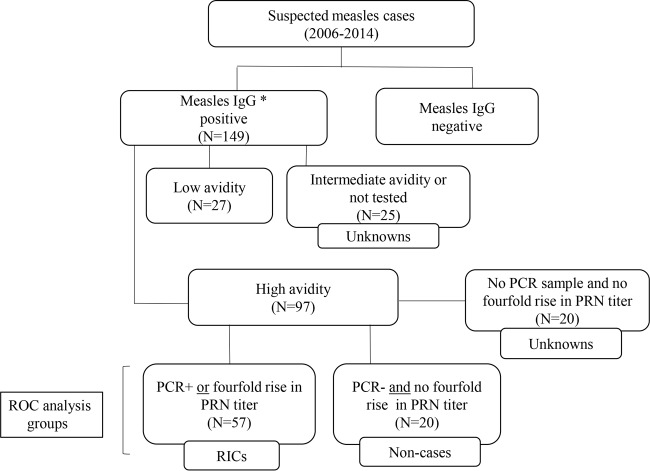
Classification scheme of RICs and noncases included in the ROC analysis and derivation of suspected cases evaluated following the ROC analysis. High-avidity measles IgG antibody in serum was the first criterion for inclusion of RICs and noncases in the ROC analysis. The RICs included in the ROC analysis were confirmed by either a 4-fold rise in serum PRN titer or a positive RT-qPCR result, while noncases had no 4-fold rise in serum PRN titer and viral specimens were negative by RT-qPCR. *, measles-specific IgG was detected either by the CDC measles IgG indirect EIA or by a commercially available measles IgG indirect EIA.

In this report, RIC is used to define a confirmed case of measles in an individual with high-avidity measles IgG, and the definition applies whether the previous immune response was elicited by vaccination or by wild-type infection. In the absence of a gold standard assay for measles virus reinfection, the criteria for inclusion of a suspected measles case as a RIC required the measurement of high-avidity measles IgG in serum and laboratory confirmation of acute measles by detection of measles virus RNA by RT-qPCR or measurement of a 4-fold rise in PRN titer between two paired serum samples (Fig. 1). A suspected measles case with high-avidity measles IgG in serum could be considered for inclusion as a noncase only if two serum samples and a companion viral specimen had been collected. The criteria for inclusion as a noncase required that the viral specimen tested negative by RT-qPCR and a 4-fold rise in titer between paired serum samples by PRN was not demonstrated.

### Measles IgG avidity determination.

Measles IgG avidity was measured in serum samples with an assay developed at the CDC by using a commercial measles IgG EIA (Captia Measles IgG; Trinity Biotech, Jamestown, NJ, USA) modified for use with the denaturing agent diethylamine as previously described (10).

### PRN assay.

Serum was tested for measles neutralizing antibody by the PRN assay (25). The World Health Organization (WHO) second international standard antimeasles serum (IS, coded 66/202, supplied by the National Institute for Biological Standards and Control, South Mimms, United Kingdom) was included to calculate the reciprocal of the 50% endpoint titer by the Kärber method. With the validation of the second WHO standard serum, PRN titers were expressed in mIU/ml. A titer of 1:8 was equivalent to a concentration of 8 mIU/ml.

### Statistical analysis.

ROC curve analysis was used to estimate the accuracy of the parameter (measles neutralizing antibody concentration) to distinguish between a RIC and a noncase on the basis of the area under the ROC curve (AUC). ROC analysis was used to establish a cutoff based on the measles neutralizing antibody concentration and to estimate sensitivity and specificity (26). The null hypothesis for sample size calculation was an AUC of ≤0.80 (moderate diagnostic accuracy) and standard error of the AUC of ≤5% (27). RICs and noncases for the ROC analyses were defined above. An initial ROC analysis was conducted with the measles neutralizing antibody concentrations obtained with the first (or only) serum sample collected after rash onset. A second ROC analysis was performed with measles neutralizing antibody concentrations from those RIC patients that had a follow-up serum sample collected ≥3 days after rash onset. If multiple follow-up serum samples were collected, the measles neutralizing antibody concentration from the earliest follow-up sample collected after ≥3 days was used.

ROC analysis was performed and graphs were prepared with MedCalc for Windows, version 13.1.1.0 (MedCalc Software, Ostend, Belgium). The significance of the differences between the groups defined in this study was determined with the t statistic for comparison of two small sample means. All tests of significance were two tailed and unpaired. A *P* value of ≤0.05 was considered significant. Geometric mean concentrations (GMCs) were determined by using the measles neutralizing antibody concentrations obtained with the PRN assay. Therefore, GMC refers to the GMC derived from PRN titers expressed in mIU/ml.

The box-and-whisker plots and comparison tests were generated with GraphPad Prism 5 software (GraphPad Software, La Jolla, CA). Outlier and extreme outlier values were identified on the basis of the standard parameters for box- and-whisker plots. The values for Q1 − 1.5 × IQR (interquartile range) and Q3 + 1.5 × IQR are the “inner” fences, and the values for Q1 − 3 × IQR and Q3 + 3 × IQR are the “outer” fences. The outliers (shown as solid circles) are between the inner and outer fences, and the extreme outliers (shown as squares or triangles) are outside the outer fences.

## RESULTS

### Measles IgG avidity testing.

Of the 149 suspected measles cases with a serum sample that tested positive for measles IgG and had sufficient volume remaining for additional testing (Fig. 1), high-avidity measles IgG antibody was detected in serum samples from 97 cases and low-avidity measles IgG antibody was present in samples from 27 cases. The remaining 25 suspected cases had insufficient sample volume to complete avidity testing (*n* = 22), or the avidity result was in the intermediate range (*n* = 3). These 25 suspected cases, which could include noncases, primary cases, and RICs, and the 27 cases with low-avidity measles IgG antibody measured in serum were excluded from the ROC analysis (Fig. 1).

Among the 97 suspected cases identified as having serum samples with high-avidity measles IgG, there were 77 (79%) cases for which a viral specimen had been tested by RT-qPCR and two serum samples were available to test for the presence of a 4-fold rise in PRN titer. Specimens from 20 of the 77 suspected cases were negative by RT-qPCR and had no 4-fold rise in PRN titer and therefore met the criteria for noncases.

Fifty-seven persons with suspected cases whose serum samples contained high-avidity measles IgG were laboratory confirmed as having acute measles by one or both of the methods required for inclusion as a RIC (Fig. 1). Forty-eight (84%) of the 57 RICs were confirmed by detection of measles virus RNA by RT-qPCR (with or without a concomitant 4-fold rise in PRN titer between paired serum samples), and the remaining 9 RICs (16%) were confirmed by determination of a 4-fold rise in PRN titer. Of the 57 RICs, 31 (54%) had an additional serum sample collected ≥3 days after rash onset. Among the original 97 suspected cases with high-avidity measles IgG detected in serum, 20 could not be classified as either a noncase or a RIC according to the study criteria and were not included in the ROC analysis.

### Laboratory and epidemiologic characteristics of RICs and noncases.

The age range of the RICs was 2 to 65 years, and that of noncases was 1.5 to 61 years (data not shown). The median age of the RICs was significantly greater (*P* < 0.0001) than that of the noncases (30 versus 10.5 years). The gender composition of the RICs was equally divided, with 29 males and 28 females, whereas there were only 6 (30%) females among the 20 noncases (data not shown). Among the RICs, the proportion of positive IgM results increased from 51 to 68% after testing of the second serum sample, while the number of IgM-positive samples decreased slightly among the noncases between the first and second samples (Table 1).

**TABLE 1:** Characteristics of suspected measles cases included in each ROC analysis with epidemiologic context described for RICs and noncases

Type case and ROC analysis (*n*)	No. (%) with PRN concn of ≥40,000^*a*^	Days^*b*^ (median)	No. (%) IgM positive^*c*^	No. (%) with known contact with case	No. (%) associated with outbreak and/or travel	No. (%) of known sources of additional cases	No. (%) with MMR vaccination^*d*^
RICs (57)							
Sample 1 (57)	18 (31)	0–11 (2)	29 (51)	35 (61)	56 (98)	5 (9)	33 (58)
Sample 2 (31)	28 (90)	3–30 (6)	21 (68)				
Noncases (20)							
Sample 1 (20)	1 (5)	0–11 (2)	9 (45)	2 (10)	5 (25)	0 (0)	17 (85)
Sample 2 (20)	1 (5)	5–42 (9)	8 (40)				

a Concentration, in mIU/ml, of measles neutralizing antibody determined by PRN assay.

b Interval, in days after rash onset, of serum collection.

c Positive by CDC measles IgM capture assay.

d Receipt of one or more doses of MMR vaccine according to case investigation.

A greater proportion of the RICs than the noncases was associated with a higher index of suspicion of measles (known contact with a person with a confirmed case of measles, investigated as part of an outbreak, or association with recent travel) (Table 1). Five (8.8%) of the RICs were known to have been the source of infection of subsequent cases of measles, whereas there were no suspected sources among the noncases. Of the RIC patients, 58% had a vaccination history of one or more doses of MMR vaccine, while the proportion among the noncases was 85%.

### ROC analysis.

**FIG 2:**
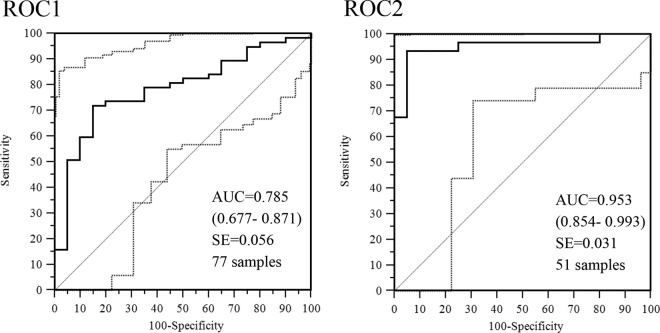
ROC curve analyses of concentrations of measles neutralizing antibodies determined by PRN assays of serum specimens from RICs and noncases. The ROC1 analysis was performed with the first (or single) sample collected. The ROC2 analysis was performed with available second samples collected from the same cases ≥3 days after rash onset. The AUC is plotted as a solid line. The diagonal line is an AUC of 0.5, interpreted as a random guess. The 95% CIs are in parentheses and are plotted as dashed lines.

The measles neutralizing antibody concentrations calculated for the serum samples from the RICs and noncases were evaluated in two separate ROC analyses (Fig. 2). The first ROC analysis (ROC1) was conducted with the measles neutralizing antibody concentrations of 77 serum samples, i.e., the first (or only) serum sample collected from 57 RICs and the first serum sample collected from the 20 noncases. The second ROC analysis (ROC2) was performed with measles neutralizing antibody concentrations of 51 serum samples that included the follow-up serum samples available from 31 of the 57 RICs and the second serum sample from the 20 noncases. In ROC1, serum samples were collected 0 to 11 days after rash onset and the accuracy with which the RICs were distinguished from the noncases was moderate (AUC = 0.785; 95% CI, 0.677 to 0.871) (Fig. 2). In ROC2, the accuracy was high (AUC = 0.953; 95% CI, 0.854 to 0.993) (Fig. 2) with measles neutralizing antibody concentrations in those serum samples collected ≥3 days after rash onset.

### Analysis of measles neutralizing antibody concentrations and identification of outliers among the RICs and noncases.

**FIG 3:**
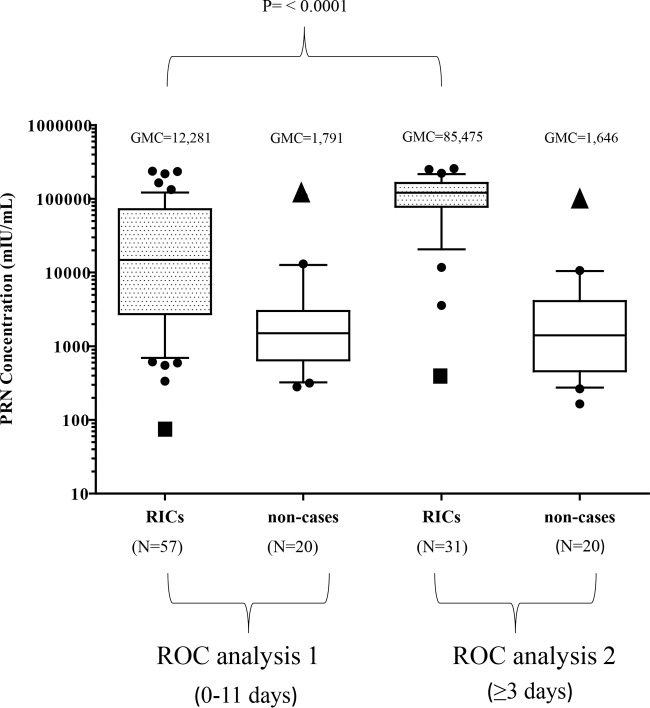
Concentrations of measles neutralizing antibodies determined by PRN assays of serum specimens from RICs and noncases used in the ROC curve analysis. Concentrations (mIU/ml) are shown as box-and-whisker (BW) plots. The whiskers represent the values within the 10th to the 90th percentiles, and the median and 25th and 75th percentiles are depicted by the horizontal lines in the boxes. Individual data points are shown; outliers are shown as black circles, and extreme outliers are shown as triangles (noncases) and squares (RICs). GMCs are shown above the BW plots. The *P* values shown are for the difference between the GMCs of serum specimens from RICs in ROC1 and ROC2. Statistical significance, *P* ≤ 0.05. N is the number of samples in each BW plot.

The GMC of the 57 RICs with serum samples collected between 0 and 11 days after rash onset (ROC1) was 12,281 mIU/ml. This was significantly different (*P* < 0.0001) from the GMC (85,475 mIU/ml) of the 31 follow-up samples (ROC2) (Fig. 3). No significant difference was observed between the GMCs of the noncases in ROC1 (1,791 mIU/ml) and ROC2 (1,646 mIU/ml).

Two data points were identified as extreme outliers in each ROC analysis. These two measles neutralizing antibody concentrations were from the same noncase (case X). The first serum sample, collected on day 3, had a concentration of 107,712 mIU/ml; the second serum sample (day 10) had a concentration of 94,860 mIU/ml (Fig. 3, triangle shapes). Although case X met the study criteria for a noncase, with no 4-fold rise and a negative RT-qPCR result, the case X patient had known contact with someone with a confirmed case of measles and was reported as having a confirmed case. However, despite the inclusion of case X as a noncase, the difference in the GMCs of the RICs and the noncases was significant (ROC1, *P* = 0.0112; ROC2, *P* < 0.0001).

### Selection of the neutralization cutoff for discriminating between RICs and noncases.

Measles neutralizing antibody concentrations of ≥40,000 mIU/ml were obtained from the first serum sample collected from 18/57 (31%) RICs and from 28/31 (90%) RICs with a second serum sample collected (Table 1). Thirty-six (63%) of the 57 serum samples evaluated in ROC1 were collected on days 0 to 2. These samples had concentrations that ranged from 81 to 236,295 mIU/ml (GMC = 5,214 mIU/ml). The concentrations of serum samples from the remaining 21 RICs, collected on days 3 to 11, ranged from 2,576 to 234,616 mIU/ml (GMC = 53,329 mIU/ml) (data not shown). Combining all of the RICs that had a first or a follow-up serum sample collected at ≥3 days after rash onset (*n* = 49), 41 (83.7%) had measles neutralizing antibody concentrations of ≥40,000 mIU/ml.

A cutoff of 40,000 mIU/ml had a specificity of 95% (95% CI, 75 to 100%) for confirmation of RICs. Furthermore, this cutoff accommodates the 3-fold variation inherent in the PRN assay (25). The specificity increased to 100% (95% CI, 82 to 100%) when two outlier values from a probably misclassified noncase were excluded (case X, discussed above) (Fig. 3). With the neutralizing antibody concentrations from serum samples collected at ≥3 days in ROC2, (*n* = 31), the sensitivity of the cutoff to identify RICs was 90% (95% CI, 74 to 98%).

### Application of the neutralization cutoff to suspected cases that were excluded from the ROC analysis.

Measles neutralizing antibody concentrations were available from the two groups that were excluded from the ROC analysis (see Fig. S1 in the supplemental material). Serum samples from 17 of the 20 suspected cases with high-avidity measles IgG antibody had at least one serum sample with a concentration of ≥40,000 mIU/ml and were designated confirmed RICs (cRICs). Eight of the 25 IgG-positive suspected cases with inconclusive avidity results (not tested or intermediate avidity) had at least one serum sample with a concentration above the 40,000-mIU/ml cutoff. Because the eight cases with concentrations of ≥40,000 mIU/ml could include primary measles cases, these cases were described as probable RICs (pRIC) (see Fig. S1 in the supplemental material). The GMCs of all of the serum samples from the 17 cRICs (130,205 mIU/ml) and the 8 pRICs (41,345 mIU/ml) were not significantly different (*P* = 0.1821) (see Fig. S2 and Table S1 in the supplemental material).

### Effect of the timing of serum collection on the presence of measles IgM and the rise in measles neutralizing antibody concentrations among the 57 RICs.

The fold increases in the PRN titers determined for 34 RICs ranged from 2- to 9,118-fold, with intervals between serum collections ranging from 2 to 29 days (see Table S2 in the supplemental material). Two of the RICs (cases 33 and 34) showed no change in titer with intervals of 7 and 18 days between sample collections. There was no correlation between the magnitude of the fold change in PRN titer and the length of the interval between sample collections, nor was there a correlation between the magnitude of the measles neutralizing antibody concentration and detection of IgM (data not shown).

**FIG 4:**
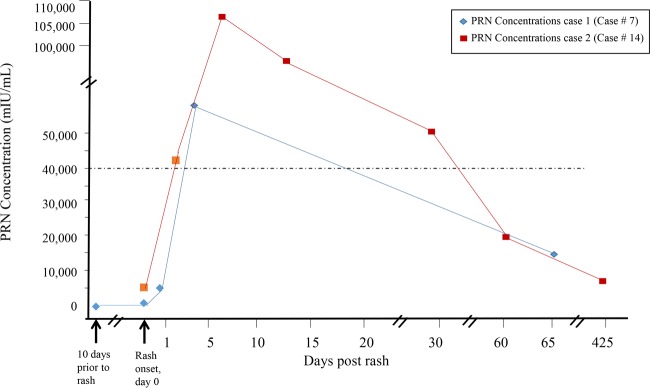
Concentrations of measles neutralizing antibodies determined by PRN assays of serum specimens from two of the RIC patients from whom multiple serum samples were collected. The graph shows concentrations of measles neutralizing antibodies and days of serum sample collection relative to rash onset.

Multiple serum samples were collected from two of the RICs (cases 7 and 14; see Table S2 in the supplemental material) included in the ROC analyses (Fig. 4). Case 7 was that of a 49-year-old health care worker who was reported to have had no detectable IgG despite the receipt of three doses of MMR vaccine. The first serum sample, drawn 1 week after exposure to a person with a confirmed case of measles, was positive for measles IgG, and the concentration was 36 mIU/ml. Case 14 was that of a 25-year-old student, born abroad, who was reported to have received one dose of MMR vaccine (undocumented) at 12 months of age. The IgG concentrations in these two RICs increased rapidly after rash onset and reached peak titers before 10 days after rash onset. Between 1 and 2 months after rash onset, the elevated concentrations had fallen below 40,000 mIU/ml (Fig. 4).

### Analysis of GMCs of noncases, RICs, cRICs, and pRICs stratified by timing of serum collection.

The GMCs of all of the serum samples, regardless of the timing of collection from the noncases (*n* = 20), RICs (*n* = 57), cRICs (*n* = 17), and pRICs (*n* = 8), were 1,385, 24,325, 130,205, and 41,345 mIU/ml, respectively (data not shown). The serum samples from these four groups were stratified into four intervals based on the timing of serum collection after rash onset for analysis of the GMCs (Table 2). The three RIC groups (RICs, cRICs, and pRICs) showed similar trends in the timing of the rise and fall of measles neutralizing antibody concentrations that were observed for two RICs with multiple serum collections (Fig. 4). Because of some low concentrations in serum samples collected ≤2 days after rash onset among the RICs and pRICs, the GMC in the noncases was not significantly different from that in the RICs (*P* = 0.1773) or the pRICs (*P* = 0.8258) for the time interval of 0 to 2 days. After 3 days, the measles neutralizing antibody concentrations increased among all of the cases identified as RICs, cRICs, and pRICs. All three of these RIC groups demonstrated a trend of decreasing concentrations at 8 days after rash onset.

**TABLE 2:** GMCs and 95% CIs of serum specimens from noncases, RICs, cRICs, and pRICS stratified according to timing of serum collection

Days^*b*^	Noncases^*a*^		RICs		cRICs		pRICs	
	No. of samples	GMC^*c*^ (95% CI)	No. of samples	GMC (95% CI)	No. of samples	GMC (95% CI)	No. of samples	GMC (95% CI)
0–2	12	1,322 (674–2,593)	36	5,214 (2,884–9,429)	5	105,714 (43,932–248,686)	3	1,904 (1,573–2,102)
3–4	4	2,859 (1,838–4,553)	16	52,819 (24,570–113,551)	4	136,888 (68,906–177,788)	3	157,012 (102,871–193,883)
5–7	9	1,838 (741–4,553)	21	102,163 (68,788–151,732)	2	197,124 (156,289–248,628)	3	200,993 (187,189–208,590)
8–42	13	951 (478–1,895)	15	57,485 (23,022–143,537)	11	130,350 (61,547–206,580)	2	55,924 (47,986–65,176)

a PRN assay-determined measles neutralizing antibody concentrations identified as outliers were excluded.

b Days are after rash onset.

c The GMCs were calculated for concentrations of measles neutralizing antibodies measured in mIU/ml.

## DISCUSSION

Laboratory confirmation of all suspected cases of measles is a critical element of surveillance and control activities during outbreaks, especially in a postelimination setting. In areas with a low prevalence of measles, the likelihood of a false-positive IgM result greatly increases regardless of the assay used. Forty-five percent of the initial serum samples submitted from the noncases in this study were positive by the CDC IgM capture assay, whereas 65% of these serum samples had previously tested positive for measles IgM by other commercial assays. Three noncases for which an etiology of rash illness was determined were attributed to parvovirus B19, enterovirus, and chikungunya virus. In the context of a febrile rash illness in a highly vaccinated population, a positive (or negative) IgM result from an acute-phase serum sample is difficult to interpret and a sample for RT-PCR may not be available. Therefore, additional assays to confirm measles reinfections may prove useful, particularly in elimination settings.

The evaluation of the RICs and noncases by ROC analysis confirmed that high concentrations of measles neutralizing antibodies can accurately identify RICs among suspected cases of measles that have high-avidity measles IgG. A measles neutralizing antibody concentration of 40,000 mIU/ml was selected as the minimum concentration for confirmation of a RIC among suspected measles cases. In this study, concentrations of ≥40,000 mIU/ml confirmed 41 (83.7%) of 49 RICs with serum collected ≥3 days after rash onset. Many of the RICs in this study had measles neutralizing antibody concentrations of ≥40,000 mIU/ml as early as the day of rash onset, which is not surprising, since exposure to virus typically occurs 12 to 14 days prior to the appearance of a rash. However, since a larger proportion of the serum samples that reached the threshold antibody concentration were collected ≥3 days after rash onset, a second serum sample may need to be tested to confirm a RIC. Collection of serum at day 3 or later is consistent with the surveillance recommendation (28) to collect a second blood sample if the first serum sample collected ≤3 days after rash onset is IgM negative or the result is inconclusive.

While many RICs may be confirmed as measles cases by epidemiologic association with another confirmed case, other situations may require laboratory confirmation. Detection of measles virus RNA by RT-PCR has become a widely available method for case confirmation, but failure to promptly collect appropriate specimens may reduce the utility of RT-PCR. Most of the follow-up serum samples in this study were late acute-phase serum samples rather than convalescent-phase samples, which are usually collected at least 2 weeks after the acute-phase serum sample for evaluation of a 4-fold rise in antibody titer. Collection of a standard convalescent-phase serum sample among suspected RICs would not be advantageous, since a rise in titer may not be observed because of the early and robust anamnestic response in most RICs. Rapid increases in measles neutralizing antibody concentrations in RICs were observed with intervals as short as 1 to 3 days between serum sample collections. However, a concentration of ≥40,000 IU/ml in a single serum sample with high-avidity measles IgG from a suspected measles case is sufficient to confirm a RIC.

Other investigators have noted the presence of high levels of measles IgG in acute-phase serum samples from measles cases in previously vaccinated individuals, although detection of IgM has varied, depending on the timing of serum sample collection and the measles IgM assay used (29–32). In the present study, 68% of the RICs that had a follow-up serum sample (*n* = 21) had a positive IgM result. Although many of the RICs in this study had early acute-phase serum samples that were strongly positive in the measles IgG EIA, individuals with rash illnesses attributable to other etiologic agents may also show strong reactivity in the measles IgG EIA as a result of immunologic stimulation and production of polyclonal IgG (33).

Measles reinfection has been described as modified measles. This term was used as early as 1963 to describe cases of measles in vaccinated individuals, as well as in patients given immunoglobulin as postexposure prophylaxis who later showed a mild clinical presentation (34). Although cases of modified measles have been characterized as having a shorter or a less severe disease course than a primary infection, some RICs exhibited symptoms that meet the clinical case definition (11, 15, 21, 35–37). Although clinical descriptions were not routinely collected, many of the RICs included in the present study were described as having mild symptoms and/or a nonclassic presentation of rash. There is evidence to suggest that individuals with a reinfection are less likely to transmit virus than a primary case of measles (18–20). However, approximately 10% of the RICs in this study were identified as the source of infection of another case. This may reflect a bias associated with RICs that are both recognized and referred to the CDC that may be different in both clinical features and the potential for transmission.

Symptomatic cases of measles in individuals who have been vaccinated have been recognized since shortly after the measles immunization program began (34, 38–40) and were described in reports of school outbreaks in the 1980s (41, 42). It is likely that many of the vaccine failures that occurred during outbreaks in the 1960s to the 1980s were mistakenly attributed to PVF because of the prevailing view that detection of IgM defined a primary immune response (30, 37, 43–46). Previously vaccinated individuals who contract measles are described as having an SVF upon the demonstration of high-avidity measles IgG. Although waning immunity (waning antibody) with time after MMR vaccination has been described (47, 48), it may not be accurate to attribute all cases of vaccine failure to waning immunity. The presence of high-avidity measles IgG antibody signifies a prior response to measles but does not provide assurance that the primary response following vaccination reached a protective titer. Immunologic mechanisms that influence both the extent of the initial response and the duration of vaccine-induced antibodies may be influenced by individual genetic differences, or other causes may contribute to incomplete immunity (49–54). Although high coverage with two doses of MMR vaccine has eliminated measles in the United States, vaccination may fail to stimulate a protective level of antibody in some individuals, even after two doses of MMR vaccine (16, 52, 55).

Eight of 74 RICs (including the cRICs) in this study were recorded as unvaccinated yet had high-avidity measles IgG. These individuals may have received a vaccination early in life, or it is possible that they had measles in childhood. Three of the RIC patients were ≥52 years of age, and two of these were born overseas in a country where measles was endemic. There are reports of individuals who have been reinfected despite natural measles in childhood; however, documentation of measles disease is not usually available and prior vaccination is difficult to rule out (11, 13, 36, 38).

This study has several limitations. Eight (14%) of the RIC patients had neutralizing antibody concentrations of <40,000 mIU/ml at ≥3 days after rash onset. The measles neutralizing antibody concentrations of these individuals ranged from 402 to 29,367 mIU/ml, and the samples were collected on days 3 to 24 after rash onset. This suggests that a proportion of RICs may not elicit a robust neutralizing antibody response regardless of the interval from rash to serum sample collection. Therefore, a neutralizing antibody concentration of <40,000 mIU/ml should not be used to rule out measles in an individual with high-avidity measles IgG that has clinically compatible symptoms.

Another limitation is that the RICs and noncases included in the ROC analyses may have been misclassified. A gold standard assay for measles reinfection does not exist. Instead, high-avidity measles IgG and either a positive RT-PCR result or a 4-fold rise in titer between paired serum samples (or both) were considered valid methods to confirm a RIC. The criteria for a noncase required both the absence of a 4-fold rise in PRN titer and a corroborating negative RT-PCR result. However, negative results by either or both assays among suspected cases with high-avidity IgG could result in misclassification of a RIC as a noncase. Indeed, a negative result by RT-qPCR and a very high measles neutralizing antibody level in the first serum sample eliminated the ability to demonstrate a rise in titer and resulted in the inclusion of a RIC among the noncases (case X). The other noncases had a low suspicion of measles and had been ruled out following extensive investigations. The presence of high-avidity measles IgG antibody in serum from the noncases would exclude the possibility of a primary infection with measles.

Finally, the noncases in our study may have had lower levels of measles neutralizing antibody concentrations than what is typical in the general population. The measles neutralizing antibody concentrations calculated for the 20 noncases (excluding a misclassified RIC) ranged from 165 to 13,083 mIU/ml (GMC = 1,790 mIU/ml), which is consistent with data from other studies, many of which measured measles antibody titers shortly after vaccination or revaccination (56–59). In one of those studies, 304 school age children who received a second dose of MMR vaccine, even those who were seronegative after the first dose, had a GMC of 2,814 mIU/ml (range, 371 to 16,400 mIU/ml) at 1 month after receipt of the second dose (56). Also, 85% of the noncases had a history of vaccination and included several individuals who had received an MMR vaccine within the previous 6 months.

The PRN assay is only available in a few reference or research laboratories, since the test requires specialized reagents and training. The need for PRN testing to confirm RICs would be limited to those settings in which endemic measles has been eliminated. Even in such settings, it is not anticipated that many suspected cases of measles would require PRN testing, since measles reinfections occur infrequently and RT-PCR is widely available. If inconclusive IgM results are obtained, RT-PCR should be utilized to confirm suspected cases of measles whenever possible. Under some circumstances, highly suspicious cases of measles that have negative RT-PCR results may require additional testing. While measles neutralizing antibody concentrations below the threshold level cannot be used to rule out a case, concentrations of ≥40,000 mIU/ml can confirm the case, albeit retrospectively. Since the PRN assay takes 7 to 10 days to complete, case investigations should not be delayed while specialized additional testing is under way. However, in an elimination setting, this additional method can aid in confirming RICs that cannot be resolved by standard techniques and have no epidemiologic link to a laboratory-confirmed measles case.

High vaccination coverage has successfully prevented measles from reestablishing endemic circulation in the United States despite continuous importation of the virus (60). As immunization programs worldwide are accelerating efforts to eliminate measles, an additional serologic method to classify challenging cases of measles may prove valuable. Further study is needed to determine if a more convenient assay format, such as EIA or microbead, can be developed or modified to generate results that correlate with neutralizing antibody concentrations in the ranges described in this study. Measles reinfections are likely to become more common when measles is introduced into highly vaccinated populations; therefore, additional data should be collected to evaluate the settings in which these cases are most likely to occur and the potential for further transmission from such cases.

## Supplementary Material

Supplemental material
