# Supplementary material for: High Concentrations of Measles Neutralizing Antibodies and High-Avidity Measles IgG Accurately Identify Measles Reinfection Cases
Source: Clin Vaccine Immunol. 2016 Aug 5;23(8):707–16. doi: 10.1128/CVI.00268-16 (PMC4979181; doi:10.1128/CVI.00268-16)
Supplement: Supplemental material [file supp_23_8_707__index.html]

Supplemental material 

# High Concentrations of Measles Neutralizing Antibodies and High-Avidity Measles IgG Accurately Identify Measles Reinfection Cases

## Supplemental material

- Supplemental file 1 -

  Legends for Fig. S1 and S2. Table S1. Description of samples and characteristics of suspected measles cases classified as either cRICs or pRICs. Table S2. PRN concentrations, IgM results, and PRN fold titer change for RICs with two serum samples collected.

  PDF, 84K
- Supplemental file 2 -

  Fig. S1. Classification scheme of suspected cases not included in the ROC analysis which were evaluated by the measles neutralizing antibody cutoff. Fig. S2. Comparison of concentrations of measles antibodies determined by PRN for serum specimens from the cRICs and pRICs.

  PDF, 16K
