# Supplementary material for: High Concentrations of Measles Neutralizing Antibodies and High-Avidity Measles IgG Accurately Identify Measles Reinfection Cases
Source: Clin Vaccine Immunol. 2016 Aug 5;23(8):707–16. doi: 10.1128/CVI.00268-16 (PMC4979181; doi:10.1128/CVI.00268-16)
Supplement: Supplemental material [file CVI.00268-16_zcd999095389so1.pdf]

Fig S1. Classification scheme of suspected cases not included in the receiver operating characteristic (ROC) analysis which were evaluated by the measles neutralizing antibody cut-off (≥40,000 mIU/mL). The cRICs (confirmed reinfection cases) included serum specimens from cases with high avidity measles IgG antibody and concentrations ≥40,000 mIU/mL. The probable reinfection cases (pRICs) had concentrations ≥40,000 mIU/mL but measles IgG avidity results were inconclusive (not available or intermediate).

Fig S2. Comparison of concentrations of measles antibodies determined by plaque reduction neutralization (PRN) (mIU/mL) for serum specimens from the confirmed reinfection cases (cRICs) and probable reinfection cases (pRICs). The geometric mean concentration (GMC) was calculated for all serum samples collected from cases in both groups. N=number of cases. The half-shaded shapes denote the concentrations of measles antibody from the first and second serum samples collected from 3 of the pRICs.

Table S1. Description of samples and characteristics of suspected measles cases (Fig. 4, two groups of unknowns) classified as either cRICs (PRN concentration ≥cut-off for RIC, high avidity IgG measles antibody) or pRICs (PRN concentration ≥cut-off, measles IgG avidity inconclusive or not done)

Designation of unknowns with PRN concentration ≥40,000mIU/mL N=45	Days[a] (median)	IgM Positive[b] (%)	Known contact with case (%)	Associated with outbreak &/or travel (%)	Known source for additional case(s) (%)	MMR history[c]
cRICS N=17	0-34 (8)	13 (76)	10 (59)	16 (94)	3 (18)	11 (65)
pRICs N=8	1-17 (4)	4 (50)	6 (75)	7 (88)	0 (0)	4 (50)

PRN, plaque reduction neutralization; cRIC, confirmed reinfection case; pRIC, probable reinfection case; MMR, measles, mumps, rubella.

[a] Interval, in days post rash onset, for serum collection.

[b] Cases with a positive IgM result by CDC measles IgM capture assay (≥1 serum sample with positive IgM result).

[c] Receipt of ≥1 dose of MMR vaccine according to case investigation.

36

37

38

Table S2. PRN concentrations, IgM results, and PRN fold titer change for RICs with 2 serum samples collected

Case #	Serum 1			Serum 2			Interval in days S1 to S2[c]	Fold titer increase or NSC
	Day[a]	PRN (mIU/mL)[b]	IgM result	Day[a]	PRN (mIU/mL)[b]	IgM result		
1	-7	12	N	10	109,423	P	17	9,118
2	1	594	N	5	192,035	P	4	323
3	2	881	N	7	119,287	N	5	135
4	0	551	N	6	66,258	P	6	120
5	2	714	N	5	84,631	N	3	118
6	0	1,367	N	4	150,219	P	4	110
7	0	1,048	N	4	56,178	N	4	54
8	0	2,253	N	10	115,664	P	10	51
9	0	8,306	N	4	249,831	N	4	30
10	2	4,898	P	14	120,578	P	12	25
11	0	2,022	P	2	50,687	P	2	25
12	2	2,884	IND	8	62,429	P	6	22
13	0	612	N	2	11,572	N	2	19
14	0	5,674	N	6	107,652	P	6	19
15	1	5,648	N	30	77,935	N	29	14
16	0	848	N	4	11,695	P	4	14
17	1	12,267	P	5	161,058	P	4	13
18	1	12,807	N	5	163,992	N	4	13
19	1	8,069	P	25	95,253	P	24	12
20	1	15,385	IND	6	144,574	P	5	9

21	1	13,032	N	5	117,169	P	4	9
22	2	16,644	P	8	123,844	P	6	7
23	0	17,066	N	22	122,306	P	21	7
24	1	36,919	IND	4	257,514	P	3	7
25	1	25,159	N	12	148,459	N	11	6
26	2	15,026	P	6	88,988	P	4	6
27	2	81	P	9	402	P	7	5
28	1	32,724	N	6	156,288	N	5	5
29	1	38,725	P	6	168,958	P	5	4
30	0	59,914	P	10	181,253	P	9	3
31	3	87,155	P	6	221,291	P	3	2.5
32	3	94,860	N	7	171,632	N	4	2
33	6	4,552	N	24	3,579	N	18	NSC
34	6	83,004	P	13	58,015	P	7	NSC

PRN, plaque reduction neutralization; RIC, reinfection case; P, positive; N, negative, N; IND, indeterminate; NSC, no significant change

[a] Day of collection; all are post rash onset except for patient #1. The S1 serum was collected after exposure to a measles case, 7 days prior to rash onset. Collection days shown with underline did not meet time interval requirement for ROC analysis (all serum samples analyzed were post-rash; second serum (ROC-2) collected at ≥3 days post rash).

[b] Concentration of measles neutralizing antibody determined by PRN in mIU/mL

[c] S1, serum 1; S2, serum 2
