# Supplementary figures and images for: High Concentrations of Measles Neutralizing Antibodies and High-Avidity Measles IgG Accurately Identify Measles Reinfection Cases
Source: Clin Vaccine Immunol. 2016 Aug 5;23(8):707–16. doi: 10.1128/CVI.00268-16 (PMC4979181; doi:10.1128/CVI.00268-16)

Classification By Measles  
Neutralizing Antibody Cutoff  
(40,000 mIU/mL)

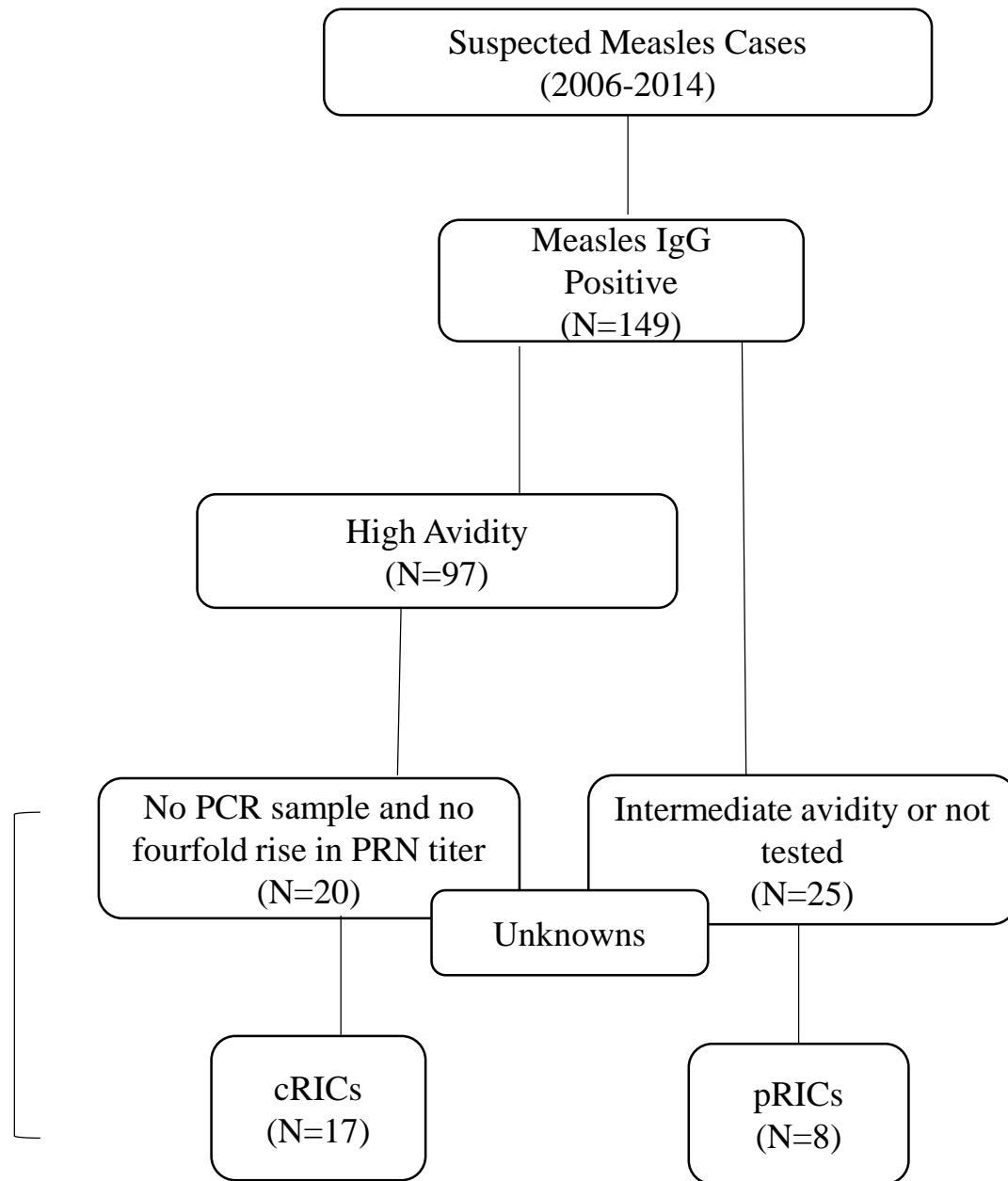

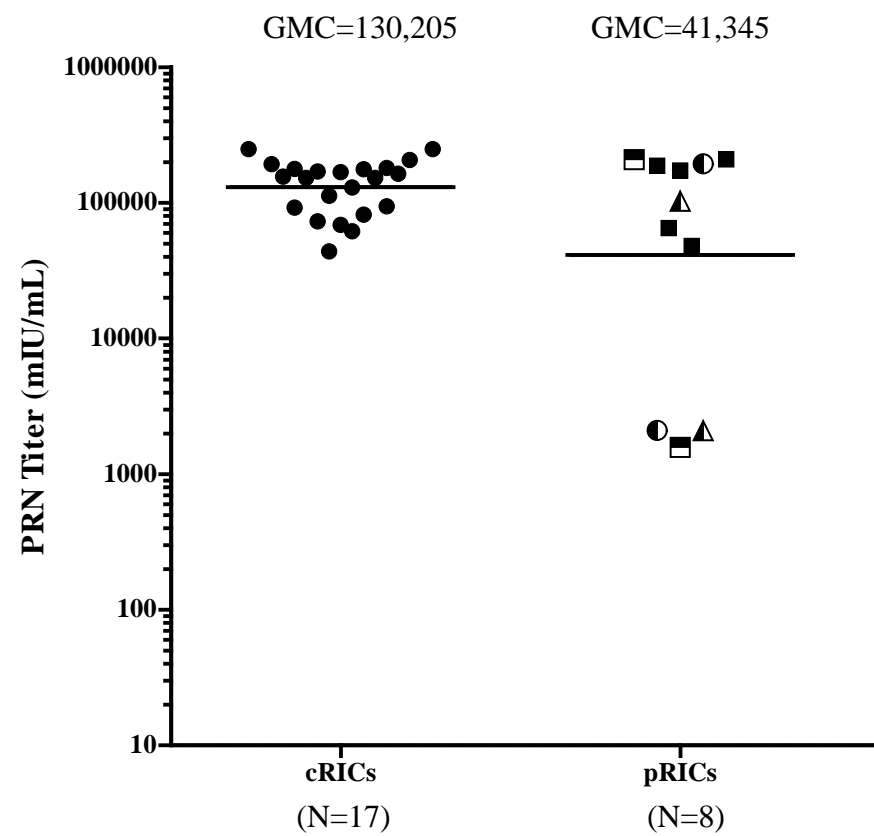

Supplement: Supplemental material [file CVI.00268-16_zcd999095389so2.pdf]
